# Motif-based community detection in heterogeneous multilayer networks

**DOI:** 10.1038/s41598-024-59120-5

**Published:** 2024-04-16

**Authors:** Yafang Liu, Aiwen Li, An Zeng, Jianlin Zhou, Ying Fan, Zengru Di

**Affiliations:** School of Systems Science, Beijing Normal University, Beijing, 100875 People’s Republic of China

**Keywords:** Complex networks, Information technology, Information theory and computation

## Abstract

Multilayer networks composed of intralayer edges and interlayer edges are an important type of complex networks. Considering the heterogeneity of nodes and edges, it is necessary to design more reasonable and diverse community detection methods for multilayer networks. Existing research on community detection in multilayer networks mainly focuses on multiplexing networks (where the nodes are homogeneous and the edges are heterogeneous), but few studies have focused on heterogeneous multilayer networks where both nodes and edges represent different semantics. In this paper, we studied community detection on heterogeneous multilayer networks and proposed a motif-based detection algorithm. First, the communities and motifs of multilayer networks are defined, especially the interlayer motifs. Then, the modularity of multilayer networks based on these motifs is designed, and the community structure of the multilayer network is detected by maximizing the modularity of multilayer networks. Finally, we verify the effectiveness of the detection algorithm on synthetic networks. In the experiments on synthetic networks, comparing with the classical community detection algorithms (without considering interlayer heterogeneity), the motif-based modularity community detection algorithm can obtain better results under different evaluation indexes, and we found that there exists a certain relationship between motifs and communities. In addition, the proposed algorithm is applied in the empirical network, which shows its practicability in the real world. This study provides a solution for the investigation of heterogeneous information in multilayer networks.

## Introduction

The research of multilayer networks is a current frontier and a hot issue in the field of complex networks, which considers multiple types of nodes and edges relationships (including intralayer and interlayer edges), reflecting the heterogeneity of nodes and edges in networks. In general, a network in which the nodes are homogeneous and the edges are heterogeneous is called a multiplexing network^1–4^, for example, in social networks, there are two different social relationships between the same users, friendship and work, which can be abstracted into different layers in networks^5,6^. At present, research on multiplexing networks has covered many aspects, such as robustness^7,8^, dynamics^9,10^, community structure^3,11,12^, disease transmission^13,14^, etc. Further, a network in which both nodes and edges are heterogeneous is called the heterogeneous multilayer network^15–17^. For example, in financial systems, the heterogeneity of nodes is reflected in the two different individuals of stocks and users, and the heterogeneity of edges is reflected in the stock relationship, the user relationship, and the relationship between the user and the stock they hold can be constituted as a two-layer network^18^. In ecological networks, the heterogeneity of nodes is reflected in the two different individuals of plants and animals, and the heterogeneity of edges is reflected in the relationship between plants, the relationship between animals, and the relationship between plants and animals^19–21^. Due to the complexity caused by the heterogeneity of nodes and edges, most of the existing studies only focus on the robustness^22,23^ and cascade failure^24^ of the network, and there are few studies focusing on community detection in heterogeneous multilayer networks.

Community structure is the main macroscopic feature of complex networks^25^. Most of the current research on the community structure of multilayer networks is based on multiplexing networks, in which the community refers to the structure consisting of homogeneous nodes that are all more tightly connected in different layers^11^. Community detection on multilayer networks is usually implemented based on three methods: the algorithms based on modularity optimization^26–28^, network layer aggregation-based algorithms^29,30^, and dynamics-based algorithms^31,32^, among which modularity is the most widely used^4,33^. In 2010, Mucha et al.^26^ summarized the previous studies to obtain a modularity function for multilayer networks. In 2018, Pamfil et al.^27^ obtained various types of multilayer networks and demonstrated its effectiveness in synthetic and empirical networks. In 2019, Zhang et al.^28^ proposed a multilayer edge mixture model, and identifies different communities. Although the existing research on community detection of multiplexing networks has been relatively mature, it does not take into account the heterogeneity of nodes.

In the heterogeneous multilayer network, the community refers to the structure consisting of heterogeneous nodes that are all more tightly connected in different layers. Compared with the community of multiplexing networks, the community of heterogeneous multilayer networks can reflect the relationship between different types of individuals in different environments. Therefore, it is very necessary to study the community detection of the heterogeneous multilayer network. At present, there are some researches about it. Lin et al. proposed MetaGraph decomposition framework to extract communities from networks containing various social backgrounds and interaction relations^34^. Liu et al. proposed the use of composite modularity for community detection of heterogeneous multi-relational networks, and realized community detection of multi-relational networks^35^. Pramanik et al. proposed the multilayer modularity index to detect communities consisting of only one type or multiple types of nodes (and edges)^36^. However, the existing research mainly explores the low-order structure by using the information of nodes and edges in the network alone, without considering the high-order structure (e.g. motif), which contains more meso-scale information about the network^37–39^.

The motif is a form of a higher-order structure, which refers to a network subgraph with a higher probability of occurrence than in a random network. The existing research shows that the use of motifs for community detection can get a better effect. Reference^39^ proposes a community detection method using motif weighted tags based on single-layer networks. Reference^37^ proposed a generalized framework for community detection based on high-order structure, and obtained good results in single layer network. Reference^38^ extended the motif to multiplexing networks. It integrated the method of network layer aggregation, used multilayer topology information to construct a single-layer network, and the results of community detection are satisfactory. However, the above studies are based on single-layer networks and multiplexing networks, which do not consider the heterogeneous multilayer networks.

In this paper, we consider the research of community detection algorithm based on motif in heterogeneous multilayer networks. First, we redefine motifs of multilayer networks to break through the problem of heterogeneity in the study of multilayer networks and provide a solution for the study of heterogeneity. Then, we modified the modularity of multilayer networks by interlayer motifs and intralayer motifs, and proposed an Motif-Based Community Detection in Heterogeneous Multilayer Networks (CDMMHN) Finally, we performed an experimental evaluation on synthetic networks. To investigate the effectiveness of the proposed algorithm, we conducted an extensive experimental evaluation on networks containing different densities of community structures, and the results show that our algorithm is effective.

The information for each section of this article is as follows. In Section 1, we summarize some related research on multilayer networks. In Section 2, we describe the problem and define the community structure of the heterogeneous multilayer network. In Section 3, we define the motifs of multilayer networks and propose a motif-based modularity that is suitable for heterogeneous multilayer networks, and propose a community detection algorithm by motif-based modularity. In Section 4, we use evaluation indicators to measure the algorithmic results of the community detection for the more general multilayer networks which are synthetic, and the relationship between the motif and community structure in multilayer networks is analyzed, and community detection was performed in empirical networks. In Section 5, we summarize our work and provide an outlook on future research directions.

## Related work

Current research for multilayer networks involves a variety of forms of multilayer networks, especially multi-relational networks. One of these classes refers to networks in which different layers of networks represent different interactions between the same individuals (nodes in different layers may be missing and increasing, but the vast majority of nodes in different layers of networks are consistent). Take two-layer social network as an example, the nodes represent users, and the connected edges refer to the network built by two kinds of relationships (e.g., interaction, following, etc.) of these users. There is a one-to-one correspondence between the nodes in different layers of the network, but there are no substantial edges, all such networks are called multiplexed networks.

At present, many researches on community detection are based on multiplex networks, in 2019, Alimadadi et al.^40^ proposed a semi-supervised joint symmetric non-negative matrix decomposition using topological information of the network as well as prior information algorithm for community detection in multilayer networks; in 2022, Venturini et al.^41^ investigated multilayer networks with the same set of nodes but without interlayer contiguous edges and proposed a filter-based multi-objective optimization approach for community detection by maximizing the modularity of different layers; in 2022, Ortiz-Bouza et al.^42^ proposed a multiple orthogonal nonnegative matrix TriFactorization method to achieve the detection of cross-layer communities in multilayer networks as well as unique communities on a single layer; in 2023, Roozbahani et al.^43^ designed a multi-relational directed network based on a semi-supervised approach for overlapping community detection; in 2023, Cai et al.^12^ proposed a graph convolution fusion model based on intralayer and interlayer information to achieve community detection for multiplexing networks. These studies are based on the topology information of the network, and some studies are based on the motif. In 2018, Pizzuti et al.^44^ proposed a motif-based community detection method based on multi-objective optimization, the main idea of which is based on the number of motifs; in 2023, Li et al.^45^ proposed a community detection algorithm for multiplexing networks based on motif awareness, which reduces the loss of information during network aggregation and improves the quality of community detection.

The other category refers to networks in which other interrelationships are added to the underlying relationships. In the case of two-layer networks, for example, a two-layer network with interlayer edges is constructed by introducing a third type of interaction between individuals (e.g., friendship) into the two-layer network as an interlayer edge of the network. In 2020, Contisciani et al.^46^ proposed a principled probabilistic approach for community detection in multilayer networks by fusing the attributes of nodes and network structure information; in 2022, Al-sharoa et al.^3^ proposed a joint non-negative matrix decomposition method for the community detection of multilayer networks by dividing the multilayer network design into a combination of multiplexing and dichotomous networks.

However, in the existing studies, there are few studies focusing on community detection in heterogeneous multilayer networks, and most of the existing studies are directed to the robustness^22,23^ and cascade failure^24^ of the network, and there are almost no studies on the community division of the network. We consider the design of a method for community detection for multilayer networks that are heterogeneous in terms of both nodes and edges.

## Community structure of heterogeneous multilayer networks

**Figure 1:**
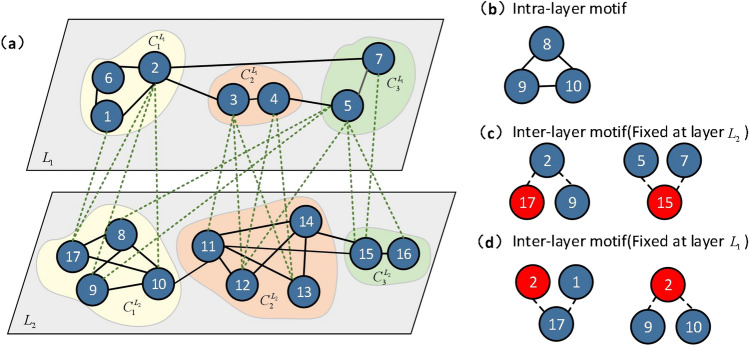
Community structure of heterogeneous multilayer networks. **(a)** represents the community structure of the multilayer network. Different colors represent different communities, which are divided into three communities ($C_{1}$, $C_{2}$ and $C_{3}$). **(b)** shows the structure of the intralayer motif for the network. **(c)** shows the structure of the interlayer motifs for the network based on layer $L_{2}$. **(d)** shows the structure of the interlayer motif for the network based on layer $L_{1}$.

In multiplexing networks, because the nodes in different layers represent the same individuals, community detection needs to integrate the information of all layers and obtain a unified community detection result of the multiplexing network. However, for the heterogeneous multilayer network, because the nodes at different layers are not consistent, it is necessary to combine the information of intralayer edges and interlayer edges to make the nodes at different layers get a unified division. As shown in Fig. 1a, taking two-layer networks as an example, layer $L_{1}$ and layer $L_{2}$ are two networks constructed based on the intralayer edges, a bipartite network can be constructed based on the interlayer edges. For this two-layer network, $G=\left\{ {G_{in} \texttt {,} G_{out}}\right\}$, where $G_{in}$ represents the set of intralayer networks and $G_{out}$ represents the set of interlayer networks. $G_{in}=\left\{ {G_{in}^{L_1} \texttt {,} G_{in}^{L_2}}\right\}$, $G_{out}=\left\{ {G_{out}^{{L_1}{L_2}}}\right\}$, where $L_1$ and $L_2$ represent different layers of the multilayer network. Because the nodes of each layer in the network represent a type of individuals, $N_{L_{1}}$ ($N_{L_{2}}$) is used to represent the nodes of layer $L_{1}$ ($L_{2}$), $E_{L_{1}}$ ($E_{L_{2}}$) is used to represent the intralayer edges of layer $L_{1}$ ($L_{2}$), and $E_{{L_{1}}{L_{2}}}$ is used to represent the interlayer edges between layer $L_{1}$ and layer $L_{2}$.

Next, the community structure of heterogeneous multilayer networks is described in detail. In Fig. 1a, different colors represent different communities. To community $C_ {1}$, it includes three parts: $C_ {1} ^ {L_ {1}}$, $C_ {1} ^ {L_ {2}}$, and the joined edges between the nodes (1, 2, 6, 8, 9, 10 and 17) of the two parts. In the layer of $L_ {1}$, the nodes (1, 2, 6) in $C_ {1} ^ {L_ {1}}$ are closely connected, and these nodes are more sparsely connected to nodes in $C_ {2} ^ {L_ {1}}$and $C_ {3} ^ {L_ {1}}$ in $L_ {1}$. In the layer of $L_ {2}$, the nodes (8, 9, 10 and 17) in $C_ {1} ^ {L_ {2}}$ are closely connected, and these nodes are more sparsely connected to nodes in $C_ {2} ^ {L_ {2}}$and $C_ {3} ^ {L_ {2}}$ in $L_ {2}$. For interlayer edges, $C_{1}^{L_{1}}$and $C_{1}^{L_{2}}$ belong to the same community, the edges between nodes in $C_ {1} ^ {L_ {1}}$ of layer $L_{1}$ and nodes in $C_ {1} ^ {L_ {2}}$ of layer $L_{2}$ are close, the edges between the nodes in $C_ {1} ^ {L_ {1}}$ and the nodes in $C_ {2} ^ {L_ {2}}$ or $C_ {3} ^ {L_ {2}}$are sparser, the edges between the nodes in $C_ {1} ^ {L_ {2}}$ and the nodes in $C_ {2} ^ {L_ {1}}$ or $C_ {3} ^ {L_ {1}}$are sparser. The same is true for the other communities ($C_{2}$ and $C_{3}$).

Most of the existing studies on community detection methods for multilayer networks are based on the edge information in the network, and rarely consider the local structure (e.g., motif) that can be constructed by the edges in the network. As a special kind of multilayer network, there have been some studies proposing community detection for multiplexing networks based on motifs^38,47^, and better results have been achieved. In heterogeneous multilayer networks, due to the heterogeneity of both its nodes and edges, simply using edges for community detection of the network can not make good use of the heterogeneous characteristics of the network. Motif, as a local network structure formed by the combination of nodes and edges, is able to interpret the heterogeneity of the network in a better way, and utilize the information of the network in a more comprehensive way. Therefore, in the next study of community detection for heterogeneous networks, we consider to do it based on motifs.

## The proposed CDMMHN method

We consider designing an algorithm to break through the heterogeneity of multilayer networks and achieve the detection of community structures in multilayer networks where both nodes and edges are heterogeneous. Because motifs can well describe the heterogeneity of edges in more general multilayer networks, this section modifies a function of motif-based modularity for multilayer networks, and proposes a community detection algorithm suitable for heterogeneous multilayer networks.

### Traditional modularity function of multilayer networks

The traditional modularity function for multilayer networks targets multiplexing networks, referring to multilayer networks with consistent nodes and different edge semantics., the modularity^27^ is calculated as:

1 $$\begin{aligned} \begin{aligned} Q = \underbrace{\sum _{t=1}^T \sum _{{i,j} \in N_{t}} \bigg (A_{ij}^t - \gamma _t \frac{d_i^t d_j^t}{2m_t}\bigg ) \delta (g_{i}^t,g_{j}^t)}_{intralayer \ modularity} + \underbrace{\omega \sum _{t=2}^T \sum _{i \in N_{t}} \delta (g_{\pi _{i}^t}^{t-1},g_{i}^t)}_{interlayer \ modularity}, \end{aligned} \end{aligned}$$

where, $A_{ij}^t$ represents the adjacency matrix of the network, *t* represents the layer of the network, $d_i^t$ represents the degree of node *i* in the network on layer *t*, $m_{t}$ represents the number of edges in the current layer (layer *t*) of the network, $\delta (g_{i}^t \texttt {,} g_{j}^t)$ determines whether node *i* in layer *t* and node *j* in layer *t* belong to the same community. If node *i* and node *j* are in the same community, $\delta (g_{i}^t \texttt {,} g_{j}^t) = 1$, and if node *i* and node *j* are in the different community, $\delta (g_{i}^t \texttt {,} g_{j}^t) = 0$. $\pi _{i}^t$ refers to the parent of node *i*, which is the node that has a relationship with node *i* at another layer, and in a multiplexing network, it refers to node *i* at another layer. $\delta (g_{\pi _{i}^t}^{t-1} \texttt {,} g_{i}^t)$ determines whether node *i* in layer *t* and its parent node (node *i* in layer $t-1$) belong to the same community. If they are in the same community, $\delta (g_{\pi _{i}^t}^{t-1} \texttt {,} g_{i}^t)=1$, and if they are not in the same community, $\delta (g_{\pi _{i}^t}^{t-1} \texttt {,} g_{i}^t) = 0$.

### Motif-based modularity function of multilayer networks

Because of the heterogeneity of edges in multilayer networks, the motif is considered to study the network structure in multilayer networks. For a heterogeneous multilayer network, the edges of the same layer are homogenous, so the motif structure in the layer is consistent with the single-layer network. However, due to the heterogeneity of the two nodes that make up the connecting edges between layers, the traditional method compresses the two-layer network and puts all edges on the single-layer network, which cannot reflect the heterogeneity of nodes in different layers and the heterogeneity of edges in the network. Therefore, we redefine the motif structure of multilayer networks with heterogeneous nodes and edges.

Triangles are a more classical higher-order structure that is often used for community detection research^45,48,49^. In 2016 Benson et al.^37^ proposed a variety of triangular subgraphs with orientations (only two structures, closed triangles and open triangles, are applicable to undirected networks) and experimentally demonstrated that triangular subgraphs are very important for social networks. In the study, we consider the three-node motif structure.

Motifs refers to subgraphs that have a much higher probability of occurring in a real network than in a random network, and for the three-node subgraph, the number of closed triangles occurring in the network is much higher than in a random network. Therefore, for the intra-layer motifs, we chose the structure shown in Fig. 1b. For the interlayer modal of the heterogeneous multilayer network, since the composition of closed triangles requires intra-layer edges, the interlayer motif chooses an open triangle structure, which means that both edges composing the motif are heterogeneous edges, which is able to show the heterogeneity of the edges. If the structure of Fig. 1b is chosen as the interlayer modifiers, the heterogeneity of the interlayer edges cannot be represented. The structure of the intralayer motif is shown in Fig. 1b, and the interlayer motif of the network comes in two forms, as shown in Fig. 1c,d. Because the nodes of the multilayer network are heterogeneous, nodes of different layers refer to different individuals, so the layer where the fixed node of the motif is selected has an impact on the structure of the motif. The network layer to which the fixed node belongs is different, and the motif structure is inconsistent. We take the red nodes in the figure as fixed nodes, in Fig. 1c structure 17-2-9 (or in Fig. 1d structure 2-17-1) is defined as a regular triplet. For the structure 17-2-9 in Fig. 1c, the fixed-node 17 and node 9 belong to layer $L_{2}$ and they have the same properties. The fixed-node 17 and node 2 belong to different layers and have different properties. Therefore, the connection structure that includes a fixed node, a node that is homogeneous to the fixed node, and a node that is heterogeneous to the fixed node is called the regular triplet (the structure 2-17-1 in Fig. 1d also applies). Besides, the structure consisting of nodes 5-15-7 in Fig. 1c (or structure 9-2-10 in Fig. 1d) is defined as an inverted triplet, in which the connection structure that includes a fixed node, two nodes that are heterogeneous to the fixed node.

In addition, for different fixed nodes, the number of nodes that can form regular triples and have heterogeneity with them is different. For example, in Fig. 1a, node 12 as a fixed node can form regular triplets with node 3 and node 4. Among them, there are two regular triples with node 3 (12-3-11, 12-3-13), and one regular triplet with node 4 (12-4-13). However, for any fixed node, the other two nodes which can form an inverted triple with the fixed node belong to different layers. For any node that has a different property from the fixed node, the number of inverted triples that can be formed with the fixed node is the same. For example, in Fig. 1a, node 12 as a fixed node can form inverted triples with node 3, node 4 and node 5. For any of them, the number of inverted triples that can be formed with a fixed node is 3 (3-12-4, 3-12-5, 4-12-5). Compared with the regular triple, the function of nodes is not discriminative, so the inverted triple is not considered in the experiment. This paper studies the community structure of multilayer networks based on the above motif structure.

In more general multilayer networks, different from the multiplexing network, the network individuals in different layers are different, there are multiple nodes in neighboring layers connected to the same node. Therefore, considering the motif can reflect the heterogeneity of the network, a calculation method of motif-based modularity for more general multilayer networks is designed. The modularity obtained based on the motif can be calculated as:

2 $$\begin{aligned} \begin{aligned} Q_{motif} = \frac{1}{2\mu _{1}} \bigg [ \underbrace{\sum _{t=1}^T \sum _{{i,j} \in N_{t}} \bigg (W_{ij}^t - \frac{w_i^t w_j^t}{2w_t}\bigg ) \delta (g_{i}^t,g_{j}^t)}_{intralayer \ modularity}\bigg ]\ + \frac{1}{2\mu _{2}} \bigg [\underbrace{\sum _{t=1}^T \sum _{i \in N_{t}} w_{i \pi _{i}^t} \delta (g_{\pi _{i}^t}^{t+1},g_{i}^t)}_{interlayer \ modularity} \bigg ], \end{aligned} \end{aligned}$$

where $\mu _{1}= \sum _{t=1}^T \sum _{{i,j} \in N_{t}} W_{ij}^t$, $\mu _{2}=\sum _{t=1 }^T \sum _{i \in N_{t}} w_{i \pi _{i}^t}$ , $W_{ij}^t$ represents the adjacency matrix based on the number of the intralayer motif of *t* layer network, *t* represents the layer of the network, $w_t$ is the motif number of intralayer that can be formed in layer *t*, $w_i^t$ represents the motif number of intralayer that node *i* can form in layer *t*. $\delta (g_{i}^t,g_{j}^t)$ determines whether node *i* in layer *t* and node *j* in layer *t* belong to the same community. If so, $\delta (g_{i}^t,g_{j}^t)=1$, and if not, $\delta (g_{i}^t,g_{j}^t)=0$. $\pi _{i}^t$ refers to the set of nodes in other layers that can form the motif with nodes *i* in *t* layer. $\delta (g_{\pi _{i}^t}^{t+1},g_{i}^t)$ determines whether node *i* in layer *t* and its parent node (the set of nodes in layer $t+1$ that can form the interlayer motif with node *i*) belong to the same community. $w_{i \pi _{i}^t}$ refers to the number of the interlayer motif that can be formed by node *i* and its parent node. For example, as shown in Fig. 1a, for node 9 in layer $L_2$, nodes 2 and 5 can form regular triplets with it. For node 5, only one regular triplet can be formed. For node 2, there are two regular triplets can be formed. Set node 9 as fixed node *i*, for the interlayer edge(2-9), $w_{i \pi _{i}^t}=2$, for the interlayer edge(5-9), $w_{i \pi _{i}^t}=1$.

### Community detection algorithm by motif-based modularity for heterogeneous multilayer networks

Next, based on the modularity function of the motifs, we design a community detection algorithm for heterogeneous multilayer networks. For a multilayer network with t layers, the network structure is represented by a matrix $$G= \left[ \begin{array} {c ccc }A_{L_{1}} &{} C_{L_{1}L_{2}} &{}\cdots &{} C_{L_{1}L_{t}}\\ C_{L_{1}L_{2}}^T &{} A_{L_{2}} &{}\cdots &{}C_{L_{2}L_{t}}\\ \vdots &{}\vdots &{}\ddots &{}\vdots \\ C_{L_{1}L_{t}}^T &{}C_{L_{2}L_{t}}^T &{}\cdots &{} A_{L_{t}}\\ \end{array} \right]$$. In the process of research, we use the two-layer network for research, and the matrix of the two-layer network can be expressed as $$G= \left[ \begin{array} {c c } A_{L_{1}} &{} C_{L_{1}L_{2}} \\ C_{L_{1}L_{2}}^T &{} B_{L_{2}} \end{array} \right]$$, where $A_{L_{1}}$ and $B_{L_{2}}$ represent the information matrix of the intralayer structure of the network in the different layer. The number of nodes in the network at layer $L_{1}$ is $n_{1}$, the number of nodes in the network at layer $L_{2}$ is $n_{2}$. And there is no self-loop between nodes. The matrix $C_{L_{1}L_{2}}$is used to represent interlayer edges of a multilayer network. Take $C_{L_{1}L_{2}}$ for example,

$$\begin{aligned} C_{L_{1}L_{2}}= \left[ \begin{array} {c c c c} c_{a_{1}b_{1}} &{} c_{a_{1}b_{2}} &{}... &{} c_{a_{1}b_{n_{2}}} \\ c_{a_{2}b_{1}} &{} c_{a_{2}b_{2}}&{}... &{} c_{a_{2}b_{n_{2}}} \\ \vdots &{} \vdots &{} \ddots &{} \vdots \\ c_{a_{n_{1}}b_{1}} &{} c_{a_{n_{1}}b_{2}} &{}... &{} c_{a_{n_{1}}b_{n_{2}}} \end{array} \right] \end{aligned}$$,

where $c_{a_{1}b_{1}}$ represents whether the node $a_{1}$ at layer $L_ {1}$ and the node $b_{1}$ at layer $L_ {2}$ have edge. If there is, $c_{a_{1}b_{1}}=1$, otherwise $c_{a_{1}b_{1}}=0$. On the basis of the edge matrix, the intralayer and interlayer motif in the network are calculated respectively, so as to obtain the network motif matrix *W* (the intralayer and interlayer motif with three nodes). $$W= \left[ \begin{array} {c c } W_{motif}^{L_{1}} &{} W_{motif}^{L_{1}L_{2}}\\ {} &{} \\ W_{motif}^{L_{2}L_{1}} &{} W_{motif}^{L_{2}} \end{array} \right]$$, where $W_{motif}^{L_{1}}$ and $W_{motif}^{L_{2}}$ both represent the intralayer motif matrix. Take $W_{motif}^{L_{1}}$ for example,

$$\begin{aligned} W_{motif}^{L_{1}} = \left[ \begin{array} {c c c c} 0 &{} w_{a_{1}a_{2}} &{}... &{} w_{a_{n_{1}}a_{n_{1}}} \\ w_{a_{2}a_{1}} &{} 0&{}... &{} w_{a_{2}a_{n_{1}}} \\ \vdots &{} \vdots &{} \ddots &{} \vdots \\ w_{a_{n_{1}}a_{1}} &{} w_{a_{n_{1}}a_{2}} &{}... &{} 0 \end{array} \right] \end{aligned}$$

represents the intralayer motif matrix obtained from the network at layer $L_ {1}$, where, $w_{a_{1}a_{2}}$ represents the number of intralayer motif obtained from the node $a_{1}$ and the node $a_{2}$ at layer $L_ {1}$. For the interlayer network, take $W_{motif}^{L_{1}L_{2}}$ for example,

$$\begin{aligned} W_{motif}^{L_{1}L_{2}} = \left[ \begin{array} {c c c c} w_{a_{1}b_{1}}^{L_{1}} &{} w_{a_{1}b_{2}}^{L_{1}} &{}... &{} w_{a_{1}b_{n_{2}}}^{L_{1}} \\ w_{a_{2}b_{1}}^{L_{1}} &{} w_{a_{2}b_{2}}^{L_{1}} &{}... &{} w_{a_{2}b_{n_{2}}}^{L_{1}} \\ \vdots &{} \vdots &{} \ddots &{} \vdots \\ w_{a_{n_{1}}b_{1}}^{L_{1}} &{} w_{a_{n_{1}}b_{2}}^{L_{1}} &{}... &{} w_{a_{n_{1}}b_{n_{2}}}^{L_{1}} \end{array} \right] \end{aligned}$$

refers to the number matrix of interlayer motif obtained from $L_ {1}$-based network, where $w_{a_{1}b_{1}}^{L_{1}}$ refers to node $a_{1}$ in the network of layer $L_{1}$ as the fixed-node, the number of interlayer motifs that can be formed by an edge of the node $a_{1}$ in the network at layer $L_{1}$ and the node $b_{1}$ in the network at layer $L_{2}$. $W_{motif}^{L_{2}L_{1}}$ refers to the number matrix of interlayer motif obtained from $L_ {2}$-based network.

We use motif-based modularity to detect communities in heterogeneous multilayer networks, the implementation process of the algorithm is shown in algorithm 1. Firstly, the motif matrix of the network *W* is obtained according to the structure matrix *G*. In the design, in order to improve the efficiency of the algorithm, we use the community detection algorithm for a single-layer network to divide the community of the network with fewer nodes (default $n_{1} < n_{2}$), the community ids obtained is denoted as $g^{L_{1}}$, $g^{L_{2}}$ can be obtained according to the relationship between the number of motifs among layers, but considering that there are few edges between layers, it is not possible to obtain the belonging community of all nodes in the $L_{2}$ layer. So we consider using the community detection algorithm for a single-layer network to get the initial community ids of nodes in the network of layer $L_ {2}$, integrating all community ids in the initial community $g ^ t = (g ^ L_ {1} {}, g ^ {L_ {2}})$. Using the original community to calculate modularity, and then transforming the community ids of nodes, updated community id and modularity (Step6 in algorithm 1). In the process of Step7 in algorithm 1, we need to reach the final result of the conditions if the modularity reaches its maximum value or the number of communities in different layers is the same. The final output $Q_{new}$ and $g_{new}$ meet the conditions. In the process, it should be noted that the community of nodes at different layers should be unified to ensure the correctness of the community results and the correlation between nodes at different layers.

**Algorithm 1:**
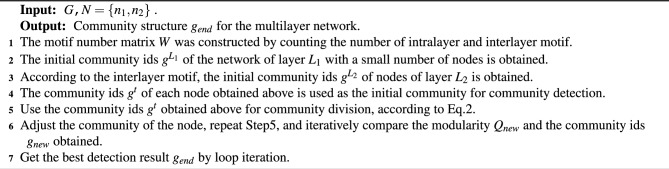
Community Detection by Motif-Based Modularity for Heterogeneous Two-layer Networks.

In the processing, there are two aspects need to be noticed. First, in the iteration, the calculation of the modularity of heterogeneous multilayer networks contains two parts, intralayer modularity and interlayer modularity. The community detection of nodes in different layers can be realized by calculating the intralayer modularity, the calculation of interlayer modularity is to unify the community of nodes of different layers, to realize the integration of multilayer information. Take a two-layer network as an example, the intralayer modularity includes the modularity calculation of the layer $L_{1}$ and the modularity calculation of the layer $L_{2}$. For the two-layer network, interlayer modularity includes modularity calculation that takes layer $L_{1}$ as the base network, changes the community number of nodes in layer $L_{2}$, and takes layer $L_{2}$ as the base network, change the community number of nodes in layer $L_{1}$. Second, because there are few edges between layers in multilayer networks, the community ids of two nodes in different layers can be obtained in the following three ways: 1) When two nodes can form the interlayer motif, these two nodes are likely to the same community. 2) When two nodes cannot form the interlayer motif, but there is an edge between them, these two nodes are likely to the same community. 3) When two nodes cannot form the interlayer motif and they are not connected, the community id of the node should be obtained according to the community ids of other nodes in the layer to which the node belongs that can form the motif with it.

## Experiment

In this section, the benchmark model is constructed for more general multilayer networks with community and we verify the algorithm on the multilayer networks. Besides, the evaluation index *NMI* and $R\_Inter$ are used to measure the accuracy of the community detection results.

### Synthetic networks

Based on the relationship between the community structure and the degree of density between nodes, this paper constructs a synthetic heterogeneous two-layer network model with a community structure. The edge density inside and outside the community within the layer as well as the effect of the specificity of fewer inter-edges on the multilayer network structure are also considered. The generation algorithm for the artificial two-layer network is shown in algorithm 2, and the network generation model is controlled by the following parameters: $c \texttt {,} n_{1} \texttt {,} n_{2} \texttt {,} z\texttt {,} z_{1} \texttt {,} p_{in} \texttt {,} p_{layer}$, where *c* is the number of communities in the two-layer network, $n_{1}$ and $n_{2}$ indicates the number of nodes at different layers in the two-layer network, *z* represents the average degree of nodes in the whole network, $z_{1}$ represents the average interlayer degree of nodes in the network, $p_{in}$ represents the probability that two nodes belong to the same community, $1-p_{in}$ represents the probability that two nodes are in different communities, and $p_{layer}$ represents the probability that two nodes belong to the same layer.

**Algorithm 2:**
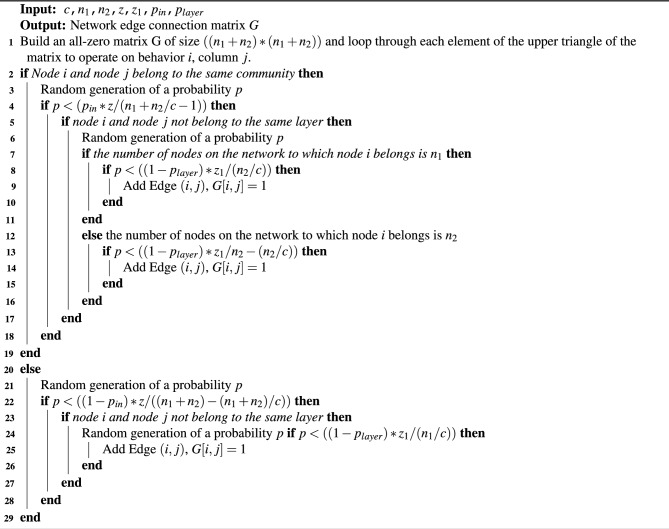
Building a benchmark model of heterogeneous multilayer networks with community structure.

During the experiment, we generated random benchmark networks with the number of communities of 3, 4, 5 and 6, the parameter settings are as follows: $c=3 \texttt {,} n_{1}=60 \texttt {,} n_{2}=90 \texttt {,} p_{layer}=0.8 \texttt {;} c=4 \texttt {,} n_{1}=80 \texttt {,} n_{2}=100 \texttt {,} p_{layer}=0.8 \texttt {;} c=5 \texttt {,} n_{1}=75 \texttt {,} n_{2}=105 \texttt {,} p_{layer}=0.8$ and $c=6 \texttt {,} n_{1}=90 \texttt {,} n_{2}=120 \texttt {,} p_{layer}=0.8$. The community to which each node belongs is fixed, and the layer to which each node belongs is determined. Once the parameter $c \texttt {,} n_{1} \texttt {,} n_{2} \texttt {,} p_{layer}$ is determined, the structure of the two-layer network only depends on the $p_{in} \texttt {,} z \texttt {,} z_{1}$.

The average degree in the network includes two parts: intra-community degree $(z_{in})$ and inter-community degree $(z_{out})$, where, $z_{in} = p_{in} * ((n_{1} + n_{2})/c - 1) / 2 \texttt {,} z_{out} = (1-p_{in}) * ((n_{1} + n_{2})/c * 2) / 2$, the average degree *z* of the network is the sum of $z_{in}$ and $z_{out}$. The degree of interlayer includes two parts: the degree of interlayer within the community and the degree between the communities of interlayer. Among them, the degree of intralayer within the community is $z_{layer}^{in}=p_{layer}*((n_{1} + n_{2})/c)/2$, and the degree of interlayer within the same community is $z_{in}-z_{layer}^{in}$; the degree of interlayer between the communities is $z_{out}*(1-p_{layer})$; the average degree of the interlayer network $(z_{1})$ is the sum of the degree of interlayer within the community and the degree between the communities of interlayer. During the experiment, we also considered the different numbers of communities in different layers, and the parameters were set as $c_{1}=3 \texttt {,} c_{2}=4 \texttt {,} n_{1}=60 \texttt {,} n_{2}=80 \texttt {,} p_{layer}=0.8$, where $c_{1}$ and $c_{2}$ represent the number of communities in different layers.

**Figure 2:**
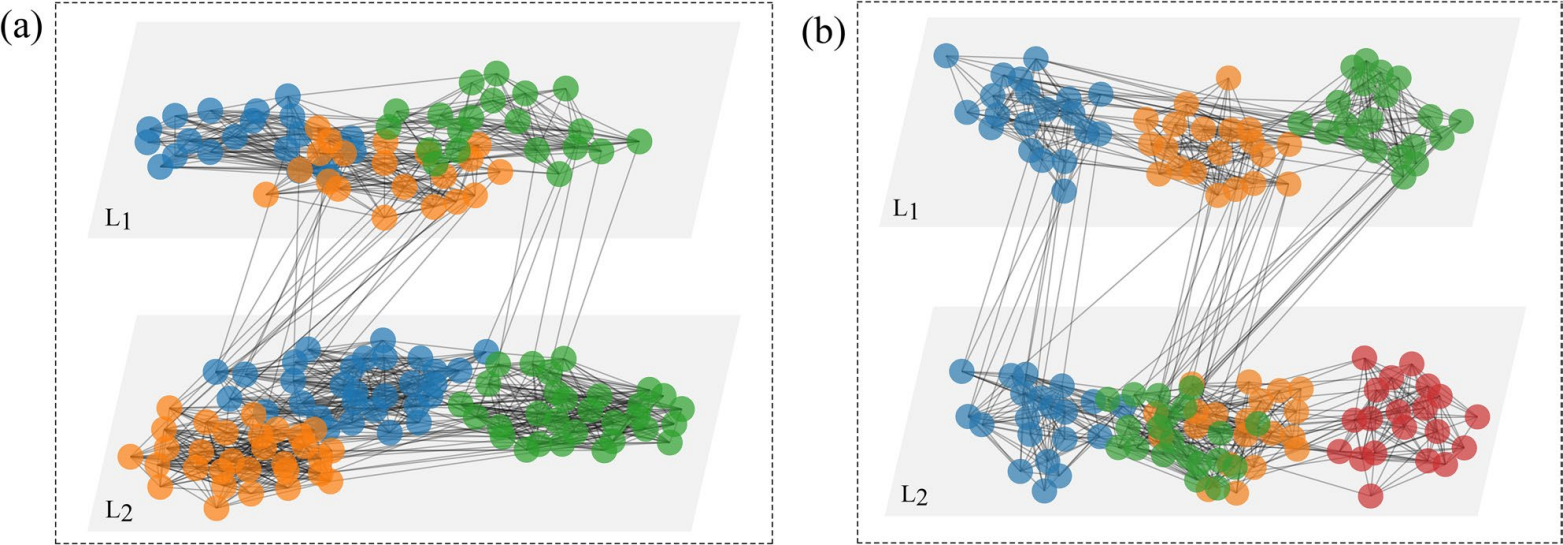
Structure diagram of a two-layer network with a known community structure. **(a)** shows a two-layer network with the same numbers of communities at different layers, and **(b)** shows a two-layer network with different numbers of communities at different layers.

The advantage of the constructed network in this way is that we can know the community id of each node in the network, which can be compared with the partition result obtained by the algorithm, so as to determine the effectiveness of the algorithm and the correctness of the partition result. According to the model of network construction, Fig. 2a is one of the network structure diagrams constructed when the parameters were $c_{1}=3 \texttt {,} c_{2}=3 \texttt {,} n_{1}=60 \texttt {,} n_{2}=90 \texttt {,} p_{layer}=0.8\texttt {,} p_{in}=0.9$, Fig. 2b is one of the network structure diagrams constructed when the parameters were $c_{1}=3 \texttt {,} c_{2}=4 \texttt {,} n_{1}=60 \texttt {,} n_{2}=80 \texttt {,} p_{layer}=0.8\texttt {,} p_{in}=0.9$.

To access the code and supplementary data used in this research, please contact the corresponding author.

### Algorithm performance

To evaluate the accuracy of the community detection results on the synthetic networks, *NMI* (Normalized Mutual Information) was used to measure the results. This metric is one of the commonly used measures of partition similarity^50^, and it can measure the accuracy of community detection as a whole. *NMI* is calculated as follows:

3 $$\begin{aligned} NMI = \frac{2I(X;Y)}{H(X)+H(Y)} \texttt {,} \end{aligned}$$

where, *X* refers to the real category. *Y* refers to the category obtained by clustering algorithm, and *H*(.) refers to the cross entropy, which is calculated as follows:

4 $$\begin{aligned} H(X) = - \sum _{i=1}^{|X|}P(i)logP(i) \texttt {,} \end{aligned}$$

*I*(*X*; *Y*) is the mutual information, $I(X;Y)=H(X)-H(X|Y)$. *NMI* is obtained by normalizing mutual information, so the range of *NMI* is [0,1]. The more similar the two vectors are, the closer the similarity is to 1. If $NMI= 1$, the result of community detection obtained by the algorithm is exactly the same as the real community IDs.

In addition, to measure the accuracy of community correspondence between individuals of different layers, we designed an evaluation index $R\_Inter$. By comparing the community ids of individuals in different layers, an interlayer correspondence matrix *C* is obtained to determine whether two nodes belong to the same community. If two nodes belong to the same community, $C_{ij}=1$, otherwise $C_{ij}=0$. $R\_Inter$ is calculated as follows:

5 $$\begin{aligned} R\_Inter=1-\frac{\sum _{i=1}^{n_{1}} \sum _{j=1}^{n_{2}} \left| C^{ij}_{real}- C^{ij}_{alg} \right| }{n_{1}*n_{2}} \texttt {,} \end{aligned}$$

where $C^{ij}_{real}$ represents the real interlayer correspondence matrix of the network, $C^{ij}_{alg}$ represents the correspondence matrix obtained by the detection algorithm, $n_{1}$ represents the number of nodes at Layer $L_{1}$, $n_{2}$ represents the number of nodes at Layer $L_{2}$, and $R\_{Inter}$ represents the ratio of correct detection in the interlayer relationship. $| C_{real}^{ij} - C_{alg}^{ij} |$ is used to determine whether node *i* belonging to layer $L_{1}$ and node *j* belonging to layer $L_{2}$ belong to the same community is correctly identified. When $| C_{real}^{ij} - C_{alg}^{ij} | = 1$, it indicateds detecting errors. Therefore, the value range of $R\_Inter$ is [0, 1]. When the interlayer community correspondence is completely correct, $R\_{Inter}=1$, when the interlayer community correspondence is completely incorrect, $R\_{Inter}=0$.

**Figure 3:**
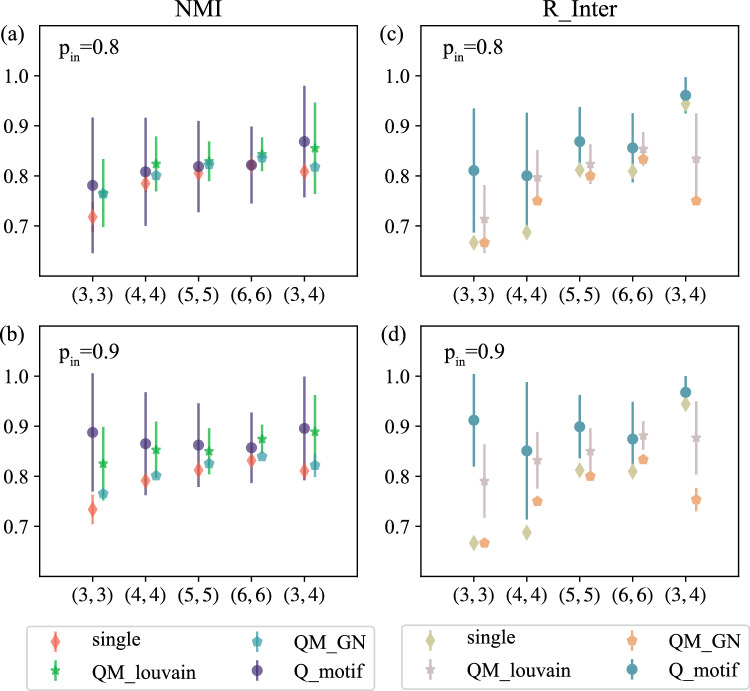
Performance of different detection algorithms in identifying community members. **(a)** and **(c)** depict the *NMI* and $R\_Inter$ values obtained based on the following parameters: $p_{in}=0.8 ~and~ p_{layer}=0.8$. **(b)** and **(d)** present the *NMI* and $R\_Inter$ values obtained based on the following parameters: $p_{in}=0.9 and p_{layer}=0.8$. The first number in the abscissa is $c_{1}$ (the number of communities at layer $L_{1}$), and the second number is $c_{2}$ (the number of communities at layer $L_{2}$).

To verify the effectiveness of the community detection algorithm in multilayer networks based on motifs, we conducted experiments on synthetic networks. At the same time, to confirm the role of heterogeneity in community detection, the two-layer network was compressed into a single-layer network, in other words, all nodes (nodes in different layers) and edges (intralayer edges and interlayer edges) were input into a network. The intralayer motif in multilayer networks is regarded as the motif of a single-layer network, which is then divided based on the motif of a single-layer network. The community detection algorithm that considers heterogeneity is compared with the algorithm that does not consider heterogeneity. To avoid the specificity of the results, we randomly generated 100 synthetic networks for the same parameters, and the results are shown in Fig. 3. The figure shows the mean and standard deviation of the accuracy of community detection for multiple experiments. $Q\_motif$ denotes the result obtained based on algorithm 1, and *single* denotes the result of partitioning the two-layer network by compressing it into a single-layer network without considering heterogeneity. $QM\_GN$^36^ and $QM\_louvain$^36^ denote the results obtained by other algorithms. Figure 3a,b provide a comparison of the overall network partition obtained with different probabilities of edges within the community, and Fig. 3c,d compare the community correspondence between nodes of different layers of the network under different edge connectivity probabilities within the community. As seen from the figure, the more obvious the community structure is, the more accurate the detected community structure is, and the nodes of different layers can be effectively unified when considering heterogeneity.

**Figure 4:**
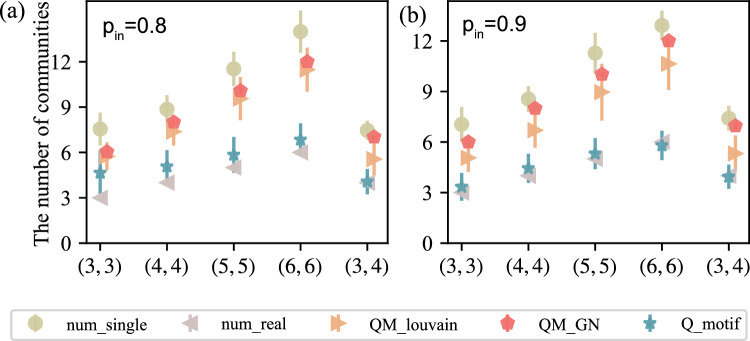
Performance of the detection algorithm in terms of identifying the number of communities. The purple triangle represents the real number of communities in the network, the yellow circle represents the number of communities detected in the single-layer network, and the blue star represents the number of communities obtained based on Algorithm 1. The red and orange colors indicate the results obtained by other algorithms.

In Fig. 3, we evaluated the accuracy of the community detection results for members of a heterogeneous multilayer network, but did not account for the accuracy of the number of communities detected by the network as a whole. Therefore, in Fig. 4, the number of communities obtained by community detection algorithms with the real number of communities are compared. The graph shows the number of communities for different probabilities of edges within a community and for different numbers of communities. In the figure, $num\_real$ represents the number of real communities in the network, $Q\_motif$ represents the number of communities detected based on Algorithm 1, $num\_single$ represents the number of communities obtained when the network is compressed to a single layer, and $QM\_GN$ and $QM\_louvain$ represent the number of communities obtained by other algorithms. As seen from the figure, with the increase in the probability of edges within a community, that is, the more obvious the community structure is, the closer the number of detected communities is to the real number of communities, and the number of communities obtained based on the algorithm is closer to the real number of communities than that obtained without considering the heterogeneity.

### Relationship between the intralayer motif and community

**Figure 5:**
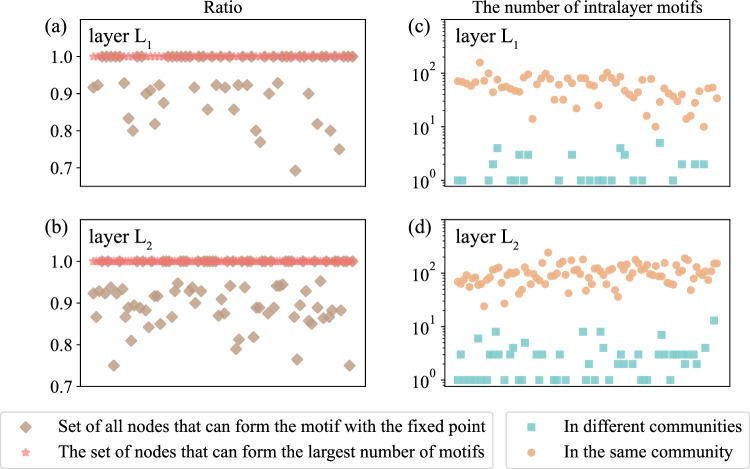
Statistical analysis of intralayer motifs and experimental results of an intralayer motif for a benchmark network with three communities.The horizontal coordinate represents each node in the corresponding network. **(a)** and **(b)** show the ratio in which motifs can be formed in layers $L_{1}$and $L_{2}$, respectively, and **(c)** and **(d)** show the number of motifs that can be formed in the same community in the same layer $L_{1}$and $L_{2}$, respectively, compared with the number of motifs that can be formed between the communities in the same layer.

In order to prove the validity of community detection using intra-motifs, we identify whether each fixed node in the layer belongs to the same community as the nodes that can form the maximum number of intralayer motifs with it and calculate the probability that the fixed node belongs to the same community as these nodes that can form the intralayer motif with it. We randomly selected one of the networks constructed based on the following parameters: $p_{in}=0.9 \texttt {,} c_{1}=3 \texttt {,} c_{2}=3 \texttt {,} n_{1}=60 \texttt {,} n_{2}=90 \texttt {,} and p_{layer}=0.8$. The results are shown in Fig. 5a,b. The brown line shows the proportion of fixed nodes that belong to the same community as other nodes in the same network that can form more than one intralayer motif, and the red line indicates the proportion of fixed nodes that belong to the same community as the other nodes in the same network that can form the largest number intralayer motifs. As seen from the figure, in this network, the fixed node must be in the same community as the nodes that can form the intralayer motif with it, and the node that has the largest number of intra-motifs must be in the same community.

In addition, we calculated the number of motifs that can be formed by nodes that belong to the same community as the fixed node and the number that are not in the same community. Taking Fig. 1a as an example, for layer $L_{2}$, the nodes that can compose the motif structure with the fixed node 11 are: 12, 14, 13, and 15. Node 11 and nodes 12, 13, and 14 are in the same community, and node 15 is not in the same community. The number of the intralayer motifs that nodes 12, 13, 14, and 15 can form is 1, 1, 3, and 1, respectively. Thus, the number of motifs that belong to the same community is 5, and the number of motifs that do not belong to the same community is 1. The statistical results are shown in Fig. 5c,d. The abscissa represents nodes, and the ordinate represents the number of motifs. The orange line is the number of motifs that can be formed within a community, and the blue line is the number of motifs that can be formed between communities. The number of motifs belonging to the same community is far greater than the number of motifs formed between nodes that are not in the same community.

### Relationship between the interlayer motif and community

In order to prove the validity of community detection using inter-motifs, we calculated the formation of regular triplets. We used layer $L_{1}$ and layer $L_{2}$ as the base layer, assumed that each node in the layer was a fixed node, and then calculated the probability that the fixed node belongs to the same community as the nodes that can form the motif with it, and the probability that the nodes that have interlayer edges with the fixed node belonging to the same community. As shown in Fig. 1a, with layer $L_{2}$ as the base layer and node 9 as the fixed node, there are two nodes (2 and 5) belonging to layer $L_{1}$ and connected to fixed node 9, one of which (node 2) is in the same community as node 9. Therefore, among the nodes with which node 9 can form an interlayer edge, the probability that node 9 belongs to the same community is 0.5. Nodes 2 and 5 form the motif with node 9. Nodes 2 and 9 can form two interlayer motifs, and node 5 can form one interlayer motif. Therefore, node 9 tends to be in the same community as node 2.

**Table 1:** Statistical analysis of interlayer edges and motif based on a network.

Network	Interlayer motif			Interlayer edges		
	Motif^1^	Same community^2^	ration	Edge^3^	Same community^4^	Ration^5^
$L_{1}$	3	3	1.0	22	20	0.9
$L_{2}$	6	6	1.0	22	20	0.9

^1^ It refers to the number of objects that can be made up.

^2^ The number of nodes that make up the motif that belong to the same community as the consent community.

^3^ It refers to the number of edges that exist between layers.

^4^ It refers to the number of nodes in the same community of two nodes with edges between layers.

^5^ ratio=same community/edge.

In a heterogeneous multilayer network, there are few edges between layers. Therefore, we analyze the statistics of the edges between layers in the whole network, and the results are shown in Table 1. The results in the table are based on one of the networks with $c_{1}=c_{2}=3 \texttt {,} n_{1}=60 \texttt {,} n_{2}=90 \texttt {,} p_{layer}=0.8\texttt {,} and~ p_{in}=0.9$. It can be seen that fixed nodes and nodes that can form interlayer modules with them are essentially in the same cluster, and most of the nodes with which they have interlayer edges belong to the same community.  Table 2 shows the mean values of the metric *ratio* in Table 1, which obtained based on multiple networks, and it can be seen that this phenomenon exists in all networks.

**Table 2:** Statistical analysis of interlayer edges and motif of different structural networks.

Network	$p_{in}$	(3,3)^1^	(4,4)^2^	(5,5)^3^	(6,6)^4^	(3,4)^5^
$L_{1}$	0.9	0.957/0.961	0.966/0.967	0.975/0.976	0.975/0.984	0.960/0.972
$L_{1}$	0.8	0.893/0.895	0.887/0.912	0.923/0.945	0.912/0.961	0.898/0.931
$L_{2}$	0.9	0.958/1.000	0.968/0.995	0.976/1.000	0.981/1.000	0.964/1.000
$L_{2}$	0.8	0.890/0.995	0.905/0.959	0.938/0.986	0.954/1.000	0.916/0.996

^1^ $$c_{1} = c_{2}=3$$.

^2^ $$c_{1} = c_{2}=4$$.

^3^ $$c_{1} = c_{2}=5$$.

^4^ $$c_{1} = c_{2}=6$$.

^5^ $$c_{1}=3, c_{2}=4$$.

### Applications in empirical networks

Finally, the algorithm is applyed to the empirical network. For an example of scientist-research topic two-layer networks, we looked at the network surrounding HQL, an academician with the Chinese Academy of Sciences (*CAS*). He is mainly engaged in condensed matter theory and related computational physics research, and his main research interests include the following aspects: strongly correlated systems, quantum entanglement and quantum phase transitions, and numerical methods for many-body systems. Based on the journal data (1893-2010) provided by the American Physical Society, we collected the (1984) papers published by Haiqing Lin’s collaborators as well as the *PACS* codes of each paper, where each *PACS* code corresponds to a specific research topic in physics.

Based on the above data, we constructed a collaborative network of scientists with Haiqing Lin’s collaborators as nodes at the scientist level. We also constructed a co-occurrence network between the *PACS* codes of the papers published by these scientists at the research topic level. In the scientist collaboration network we constructed, there are 844 nodes and 146817 edges, the nodes in the network represent Haiqing Lin’s collaborators, and the edges represent the two scientists who have coauthored at least one *APS* journal paper. In the research topic co-occurrence network, which contains 78 nodes and 241 edges, the nodes in the network represent the *PACS* codes included in the *APS* journal papers published by the above scientists, and the edges represent that the two *PACS* codes have co-occurred at least once in the *APS* journal papers published by these scientists. At the same time, based on the information that a scientist has published a paper in a particular *PACS* code, we built an edge between the scientist layer and the research topic layer. Finally, we obtained a scientist-research topic two-layer network with heterogeneous nodes and edges. Based on algorithm 1, we performed community detection on this heterogeneous scientist collaboration network, and the whole network was divided into 4 groups, which allowed us to determine in what research areas scientists mainly collaborate with each other.

If community detection for single-layer networks is performed on the scientist cooperation network and the research topic co-occurrence network, we can determine which scientists cooperate more closely with each other and which research fields are more closely connected, but we cannot integrate the scientist groups and research fields. To fuse information about scientists and research fields on top of the above two single-layer networks, it is also necessary to add information about which papers scientists collaborated on and in which papers research fields co-occur, i.e., to construct a bipartite network between scientists and papers, and a bipartite network between research fields and papers. Our approach is able to directly relate scientists and research fields together to achieve a more rational division of scientists and their research topics.

## Conclusion and future work

In this paper, the community detection algorithm of more general multilayer networks in which both nodes and edges contain heterogeneity are proposed. Among, the motif structure are used to calculate the modularity of multilayer networks, which can distinguish the intralayer and interlayer edges of networks well. We used two metrics ($NMI,R\_Index$) to measure the accuracy of community detection in terms of the overall distribution of communities and the integration of communities between different layers. The results of proposed algorithm in synthetic networks showed the more accurate community structure (the number of communities and the community to which members belong), comparing with other community detection algorithms.

We applied algorithm 1 on an empirical network, which enables the problem of detecting communities uniformly between different types of individuals. Finally, this paper analyzed the relationship between the intralayer motifs (interlayer motifs) and communities and further explains the feasibility of the algorithm.

This study provides an understanding of the community structure of more general multilayer networks, and our algorithm mainly addresses the problem of community detection in undirected multilayer networks. In the following research, we hope to conduct more in-depth research in more multi-level empirical networks. Moreover, there are many unequal relationships in real networks, such as directed networks. In the future, we will conduct an in-depth study of community detection in multilayer-directed networks.

## Data Availability

The APS data can be downloaded at https://journals.aps.org/datasets.
